# Homeopathic medication for seasonal allergic rhinitis—a randomised placebo-controlled trial

**DOI:** 10.3389/falgy.2026.1815934

**Published:** 2026-07-17

**Authors:** J. Siewert, H. Cramer, M. Ortiz, T. Tissen-Diabaté, S. Roll, K. Wegscheider, K. Linde, T. Reinhold, S. N. Willich, M. Teut, B. Brinkhaus

**Affiliations:** 1 Institute of Social Medicine, Epidemiology and Health Economics, Charité – University Medical Center Berlin, Corporate Member of Freie Universität Berlin and Humboldt-Universität zu Berlin, Berlin, Germany; 2 Institute of General Practice and Interprofessional Care, and Bosch Health Campus, Robert Bosch Centre for Integrative Medicine and Health, University Hospital Tübingen, Stuttgart, Germany; 3 Institute of Medical Biometry and Epidemiology, University Medical Centre Hamburg, Hamburg, Germany; 4 TUM School of Medicine and Health, Institute for General Practice and Health Services Research, Department of Clinical Medicine, Technical University Munich, Universität München, Munich, Germany

**Keywords:** complemantary and alternative therapies, double-blind, homeopathic medication, placebo, randomised controlled trial, rhinitis

## Abstract

**Background:**

The study aim was to evaluate the efficacy of homeopathic medication in SAR patients.

**Methods:**

In a double-blind, three-arm randomised trial, patients at eleven outpatient clinics and two medical centres were randomised to receive (1) individualised homeopathic case taking (IHCT) and standardised homeopathic medication with Galphimia Glauca (GG), (2) IHCT and individualised homeopathic treatment (IHG), or (3) IHCT and placebo (PG). Primary outcome was disease-specific quality of life, assessed using the Rhinitis Quality of Life Questionnaire (RQLQ) after three and four weeks. Secondary outcomes included response rate (≥0.5-point change in RQLQ), rescue medication use, and total nasal and non-nasal symptom scores (TNSS, TNNSS).

**Results:**

Sixty-two SAR patients (mean age ± SD: 46.9 ± 14.9; 43.5% female) were recruited, approximately 25% of the planned sample size. After weeks three and four, there were no significant differences in RQLQ (*p* = 0.244) between GG (adjusted mean, 1.2, 95% CI 0.7–1.7), IHG (1.7, 1.2–2.3), and PG (1.4, 0.8–2.0). High response rates were observed (GG: 86.4%, IHG: 66.7%, PG: 81.3%), while RM use was 21.7%, 55.6%, and 29.4%, respectively. There were no relevant differences in RM score, TNSS and TNNSS between the three groups. Eight adverse events but no serious adverse events were reported.

**Conclusion:**

Standardised and individualised homeopathic drugs were not superior compared to placebo suggesting that treatment response was not based on study medication. The validity of the study and its conclusions are limited by the fact that the recruitment target was not achieved.

**Trial registration:**

This study has been registered in the German Clinical Trial Registry with trial ID DRKS00018081.

## Background

With a prevalence between 10% to 40% worldwide, seasonal and perennial allergic rhinitis (AR) is a global health problem (1) with an estimated direct cost of 1.5 billion Euro (2). Specific immunotherapy, the only causal treatment currently approved for seasonal allergic rhinitis (SAR), is time-consuming, costly, and not effective in all SAR patients (3). Antihistamines and other antiallergic drugs do not always achieve clinically relevant symptom reduction and may cause side effects, and a large number of AR patients turn to complementary medicine treatments. In Germany, approximately one quarter of atopic diseases patients including AR use complementary therapies, of which homeopathy is the most frequently used method at 35% (4).

There is an ongoing debate regarding the efficacy of homeopathic drugs in general and many doctors and scientists strictly deny the possibility that homeopathy drugs can work (5–7). The lack of a generally accepted mechanism of action and methodological concerns regarding parts of the existing evidence base have contributed to this ongoing controversy. Results of previous published systematic reviews have been inconclusive and contradicting (8–11). However, there is some evidence that homeopathy and especially a homeopathic preparation of Galphimia glauca (a plant from the botanical family Malpighiaceae, native to Central America/Mexico) showed effects in allergic rhinitis (12, 13).

In order to overcome the existing evidence gap, well-conducted randomised controlled trials using gold standard methods such as placebo control and blinding are needed (13).

Therefore, the aim of the Homeopathy in Seasonal Allergic Rhinitis (HOMEOSAR) trial was to investigate the efficacy of individualised homeopathic case taking (IHCT) and Galphimia Glauca (GG) vs. IHCT and individualised homeopathic treatment (IHG) vs. IHCT and placebo on disease specific quality of life in patients with seasonal allergic rhinitis (SAR).

## Methods

### Study design and registration

**Figure 1:**
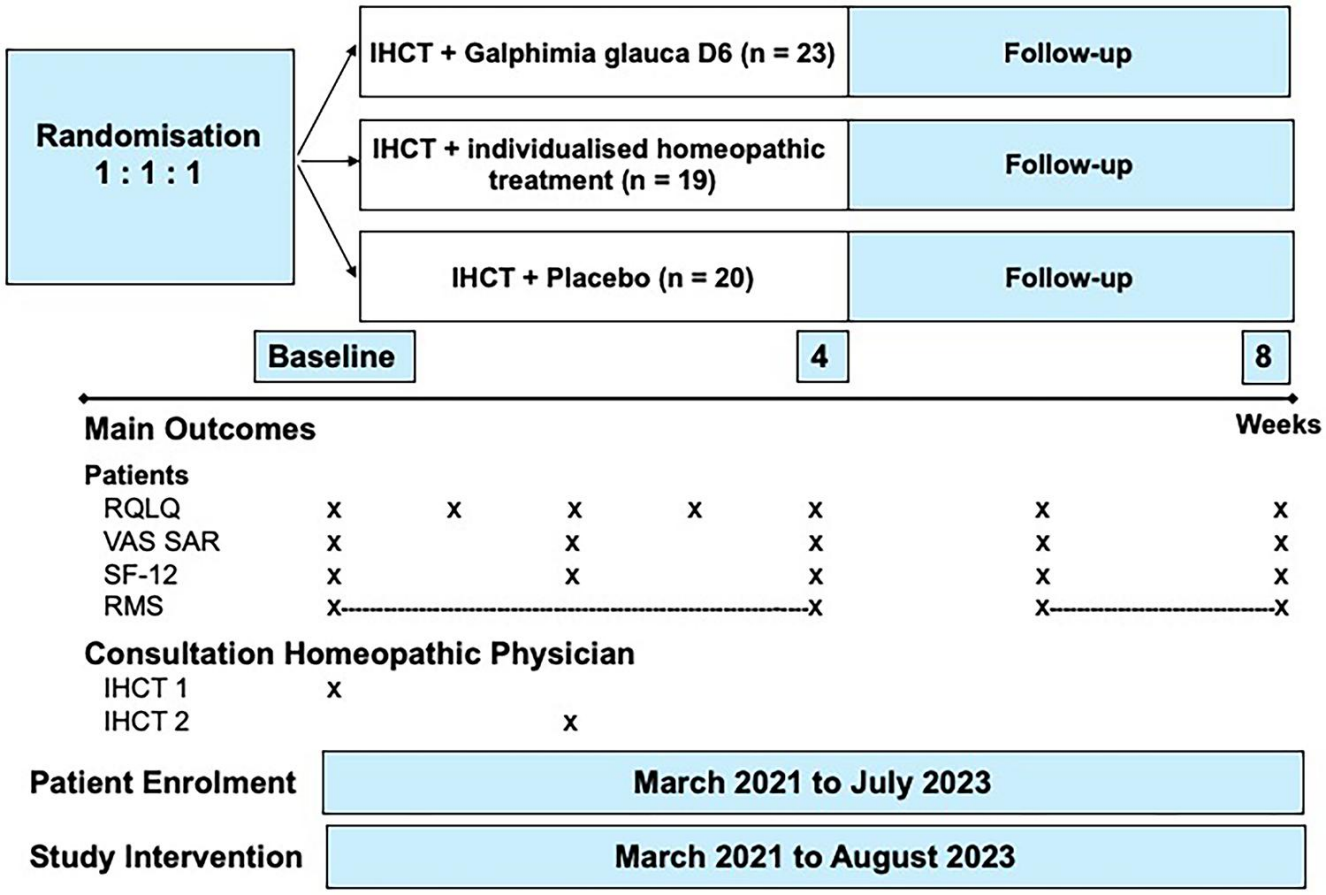
Design of the HOMEOSAR study. IHCT1, first individualized homeopathic case taking; IHCT2, second individualized homeopathic case taking; D, decimal potency; RQLQ, Rhinitis Quality of Life Questionnaire; VAS SAR, Visual Analogue Scale for seasonal allergic rhinitis symptoms; SF-12, 12-Item Short Form Health Survey; RMS, rescue medication score.

The HOMEOSAR study was a three-arm, parallel group, placebo-controlled, randomised, double-blind, multicentre trial comparing the efficacy of (1) IHCT and standardised homeopathic medication with GG, (D6 potency) vs. (2). IHCT and IHG (D6 potency) vs. (3) IHCT and placebo (placebo group, PG) in SAR patients (Figure 1). The study participants, study physicians, principal investigator, sponsor, study nurse, data manager, and data analyst were blinded to the study treatment. Design and methods of the study protocol have been described earlier in detail elsewhere (14). This study was performed according to guidelines for clinical trials (Declaration of Helsinki, Good Clinical Practice). Reporting follows the CONSORT statement (15). All study participants provided written, informed consent. The study protocol was approved by the responsible ethics committee (LaGeSo Berlin), the German BfARM (Federal Ministry for Drugs and Medical Devices; 61-390-4043945) and was registered in EUDRACT (2019-003255-10). Three amendments were submitted and approved during the trial, mainly because of security measures, restrictions during the COVID-19 pandemic situation, and minor changes in the inclusion criteria due to problematic recruitment (14).

### Patients and recruitment

Patients aged 18–75 years and diagnosed with moderate to severe SAR were included in the trial. The inclusion criteria included a skin prick test and/or a Radio-Allergo-Sorbent Test (RAST) positive for both birch and grass pollen; a score between 40 mm and 80 mm on a visual analogue scale (VAS) for the average intensity of SAR symptoms in the week prior to inclusion; an indication for the use of oral antihistamines as anti-allergic medication; and written informed consent. Important exclusion criteria included other types of chronic rhinitis; history of anaphylactic reactions; moderate to severe atopic dermatitis; and symptoms of asthma in accordance with the Global Initiative for Asthma symptom control criteria (16). For further exclusion criteria please see the protocol publication (14).

The recruitment of study participants was carried out primarily using information material at general practitioners' clinics, and advertising on public transport und in newspapers. Patients were initially screened by phone and then referred to a study physician for complete patient enrolment.

### Study procedure and setting

The study was conducted during the birch and/or grass pollen season (usually between February and August in Germany). After providing written informed consent and before randomisation, all patients included in the study received a baseline assessment and subsequently a first homeopathic consultation including IHCT (60–120 min). After the consultation, study physicians prescribed an individualised homeopathic drug for each patient, regardless of treatment group allocation. The prescription was sent by fax to the study pharmacy, where the patient was randomised to one of the three treatment groups. Based on this description and the ACCESS database, the study pharmacy allocated the patients into one of the treatment groups following the randomisation code. Patients were randomised in a 1:1:1 ratio using centre-stratified block randomisation with randomly varying block lengths. The group allocation was known only to the pharmacist and one independent unblinded data manager who was not further involved in the study organisation. Following the randomisation process, patients obtained their assigned study medication by post for the duration of the study.

The second homeopathic consultation (max. 30 min) was conducted for all patients approximately 14 days after the first consultation. Again, regardless of the blinded treatment group allocation, study physicians may have prescribed another individualised homeopathic drug. A change in the homeopathic drug was, however, only implemented for those patients randomised to IHG. Patients in the other two treatment groups continued their respective treatment, however received a new bottle of medicine from the pharmacy.

### Study interventions

Study intervention was prescribed by study physicians in their outpatient clinics or two university outpatient clinics over a four-week period in two consultations. All study physicians were specialised in conventional medicine, for example as specialists in internal medicine or general practice, and had an additional qualification in homeopathic treatment, and at least five years of practical experience providing homeopathic treatment.

Patients received (1) IHCT and standardised homeopathic medication with Galphimia Glauca (GG, dilution D6 potency), or (2) IHCT and individualised homeopathic treatment (dilution D6 potency), or (3) IHCT and placebo. IHCT included a follow-up consultation after approximately 14 days. Individualised homeopathic treatment was defined as any homeopathic medicinal product that is registered, monographed, and available from “Deutsche Homöopathie-Union” (manufacturers of homeopathic medicinal products) for prescription. To ensure blinding, placebo preparations were visibly indistinguishable from the study medication. All patients received their study medication as alcoholic dilution in identical bottles. The standard dose was five drops of the assigned study drug taken orally three times a day. Galphimia glauca D6 and all individualised homeopathic products with a potency of D6 are registered and routinely used “over the counter” drug products in Germany.

Patients in all three study arms were allowed to take up to a maximum of two 10 mg doses of second-generation oral antihistamines (i.e., cetirizine dihydrochloride) daily as rescue medication (RM) on demand. The on-demand RM (cetirizine) was included as an outcome to better monitor the use of RM and for comparison reasons when considering outcomes in the GG vs. IHG vs. PG. If the clinical symptom(s) of SAR could not be managed sufficiently by the antihistamine treatment, medication with an oral corticosteroid could be used. The use of any other anti-allergic medication was discouraged.

### Outcome measures

The primary outcome was rhinitis-specific quality of life, measured as the mean of the Rhinitis Quality of Life Questionnaire (RQLQ) overall scores in weeks 3 and 4 (17). The RQLQ mean of weeks 3 and 4 was chosen to allow enough time for the study interventions to show a potential effect, as a treatment duration shorter than three weeks was considered insufficient. In addition, averaging the RQLQ scores of weeks 3 and 4 was intended to compensate for weekly fluctuations in pollen exposure and SAR symptoms. Using the mean of both assessments was expected to provide a more stable estimate of treatment effect than a single assessment time point.

Secondary outcomes were measured at the end of weeks 3, 4, 7, and 8, and included the RQLQ overall score [range 0 (no impairment) to 6 (worst impairment)], the seven RQLQ domain scores, the nasal and the non-nasal symptoms measured by the Total (Non-) Nasal Symptom Score [TNSS (range 0–12) and TNNSS (range 0–12)], and the rescue medication. The responder status was defined as at least a 0.5-point decrease (improvement) in the RQLQ overall score (mean of weeks 3 and 4) compared to the baseline value. RM usage was scored daily between baseline and end of week 4 and in weeks 7 and 8 using the Rescue Medication Score (RMS) on a 4-point scale: no RM (0 points); cetirizine 10 mg/day or equivalent (1 point); cetirizine 20 mg/day or equivalent, (2 points); any form of systemic corticosteroids for SAR (3 points). As a *post-hoc* outcome, use of any rescue medication during the 4 weeks of intervention was regarded.

In addition, patient questionnaires included visual analogue scales (VAS, 0–100 mm) for overall SAR symptom severity and for nasal, eye, and oral symptoms, and the German version of the quality of life questionnaire SF-12 with a mental and physical health component (18). After 8 weeks, patients were asked in which group they think they were randomised.

Safety data (adverse events (AE) and serious adverse events (SAE) were collected from baseline until completion of the study. As a further secondary outcome, the impact on patients' costs was assessed; these results will be presented in a separate publication.

### Statistical analysis

The sample size calculation was described in detail in in the published study protocol. To estimate the sample size for the study, 80% power and a minimal clinically important difference (MCID) of 0.5 points (19) in the RQLQ between either one of the two verum groups and the placebo group were assumed. The common standard deviation was estimated at 1.1 (20), corresponding to an effect size of d = 0.45. With these assumptions and an assumed drop-out rate of approximately 15% (20), we planned to include 270 patients, 90 in each group.

Analyses were performed in R (21) for two analysis populations (1). A full analysis set (FAS) is based on the intention-to-treat (ITT) principle, including all randomised patients regardless of whether the intervention and other trial procedures were performed according to the protocol. Every patient was analysed as if they had conducted the trial according to the group to which they were originally allocated (2). A per-protocol population (PP) included only patients with no major protocol deviations, interventional drug intake on at least 50% of study days, no unauthorised treatment. In general, missing values were not replaced.

A closed testing procedure (22) was used for the primary outcome to handle the pairwise comparisons between the three treatment groups to control the overall type I error and to maintain the global significance level of *α* = 5% (two-sided). First, a global test was used across all three treatment groups to test the null hypothesis of equal means. If this null hypothesis was rejected, pairwise comparisons (IHG vs. PG, GG vs. PG, GG vs. IHG) were tested confirmatively. The analysis was performed on the FAS using an analysis of covariance (ANCOVA), in which the endpoint was modelled as a function of treatment group (GG/IHG/PG; fixed factor), baseline RQLQ score (linear covariate), and the study centre (random factor) to obtain the treatment group means, mean treatment group differences, respective 95% confidence intervals, and the (two-sided) *p*-values. In a sensitivity analysis, missing values in the primary outcome were imputed using multivariate imputation by chained equations (MICE).

Secondary endpoints were analysed exploratively using the FAS, with selected endpoints also assessed in the PP population. Continuous endpoints were examined via ANCOVA. Responder proportions were compared descriptively and via logistic regression, adjusting for baseline RQLQ and study centre (random factor). Sensitivity analyses modelled the primary endpoint with study region instead of study centre. Subgroup analyses considered SAR severity, sex, and treatment expectation. Further details are available in the published study protocol (14). Safety endpoints were analysed descriptively for the FAS.

## Results

**Figure 2:**
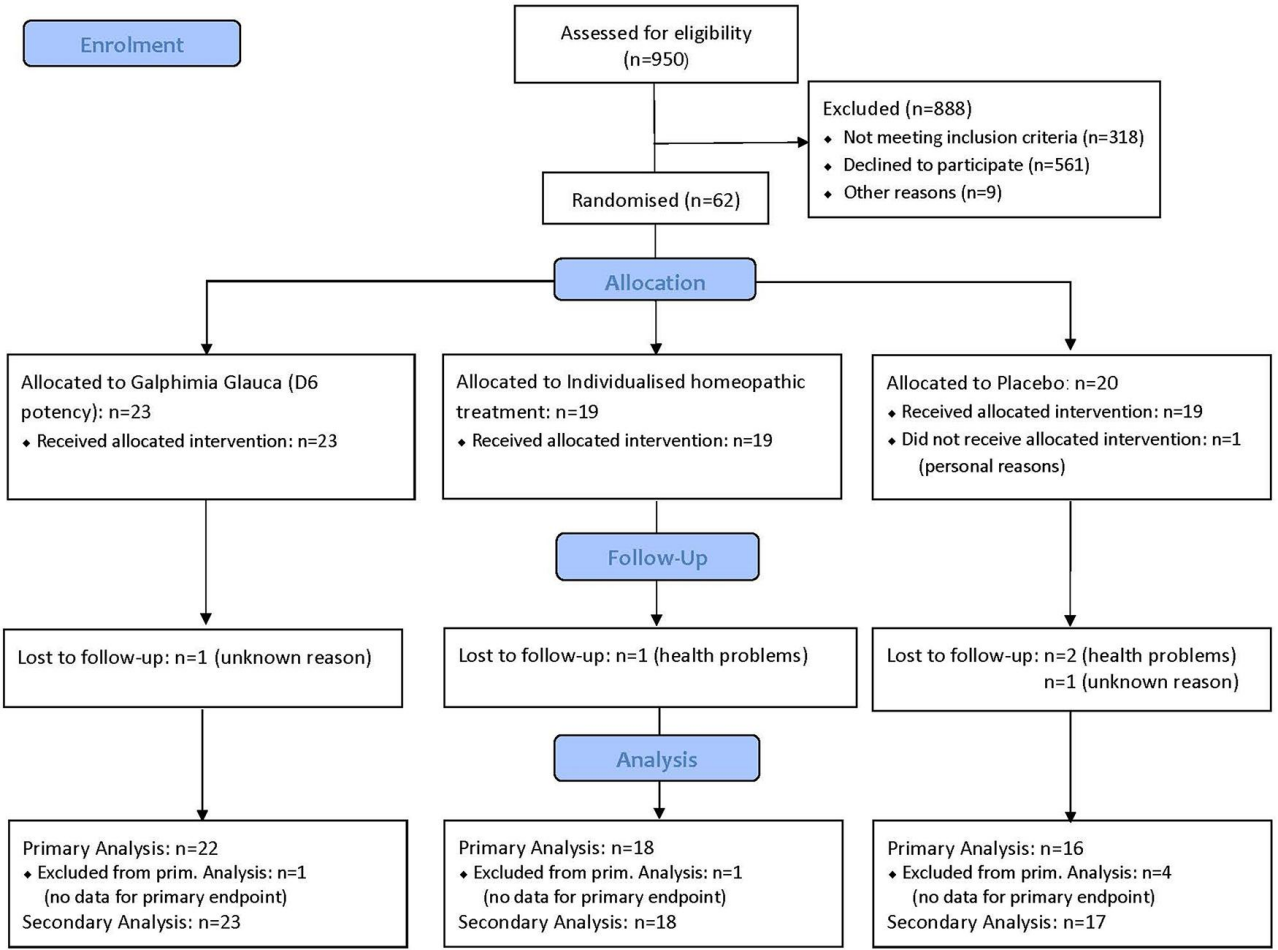
HOMEOSAR study—CONSORT flow chart.

Between March 2021 and July 2023, a total of 950 SAR patients were assessed for eligibility to participate in the study; most patients declined to participate (*n* = 561) or did not meet the inclusion criteria (*n* = 318) (Figure 2, Supplementary Table S1).

Even though the recruitment period was extended by one year, only 62 patients were included (GG *n* = 23, IHG *n* = 19, and PG *n* = 20). Data for the main efficacy analysis were available for 56 patients. Six patients (GG: *n* = 1, IHG: *n* = 1, PT: *n* = 4) were excluded from the primary endpoint analysis because of missing data for the RQLQ (Figure 2). Baseline characteristics were mainly comparable between the three study groups, however patients in the IHG were more often male, of younger age, with more anti-allergic medication, less expectations for homeopathy efficacy in SAR especially for Galphimia glauca, and higher expectation for individualised homeopathy (Table 1). Altogether, 21 patients were excluded from the PP-analysis resulting in 41 patients for PP-analyses.

**Table 1:** Baseline characteristics. Values are absolute numbers (*n*), column percentages or means and standard deviations.

Characteristics	All Patients	Galphimia glauca	Individualised homeopathy	Placebo
	(*n* = 62)	(*n* = 23)	**(*n*** **=** **19)**	**(*n*** **=** **20)**
Female, *n* (%)	27 (43.5%)	11 (47.8%)	7 (36.8%)	9 (45.0%)
Age, mean (SD), year	46.9 (14.9)	47.2 (14.2)	42.6 (14.0)	50.8 (16.0)
BMI, mean (SD), kg/m^2^	23.9 (4.2)	25.2 (6.1)	23.3 (2.2)	22.9 (2.3)
Duration of SAR symptoms, mean (SD), y	25.2 (12.3)	25.2 (10.6)	25.5 (12.8)	24.9 (14.2)
Years with SAR diagnosis, mean (SD), y	21.6 (11.8)	22.4 (10.3)	24.7 (12.8)	17.7 (11.8)
Concomitant asthma disease, *n* (%)	8 (12.9%)	2 (8.7%)	3 (15.8%)	3 (15.0%)
Concomitant eczema, *n* (%)	6 (9.7%)	0 (0.0%)	4 (21.1%)	2 (10.0%)
Prior CAM treatment due to SAR, *n* (%)	13 (21.0%)	3 (13.0%)	4 (21.1%)	6 (30.0%)
Medication due to SAR symptoms, *n* (%)	39 (62.9%)	13 (56.5%)	15 (78.9%)	11 (55.0%)
RQLQ overall score, mean (SD)^a^	2.9 (1.0)	2.9 (1.0)	2.9 (0.9)	2.9 (1.1)
SAR symptoms last 7 days, mean (SD), mm^a^				
Total SAR symptoms (VAS)	59.9 (11.7)	60.7 (12.1)	59.6 (11.8)	59.4 (11.7)
Nasal SAR symptoms (VAS)	61.1 (18.5)	62.2 (17.1)	60.2 (20.5)	60.9 (18.9)
Eye SAR symptoms last (VAS)	54.7 (23.7)	58.0 (22.7)	53.7 (23.5)	51.9 (25.9)
Respiratory SAR symptoms (VAS)	29.1 (25.3)	27.4 (23.6)	30.8 (25.3)	29.6 (28.0)
Oral SAR symptoms (VAS)	33.0 (23.9)	34.1 (24.3)	31.6 (25.9)	33.2 (22.8)
TNSS, mean (SD)^a^	8.2 (2.4)	8.7 (2.2)	8.2 (2.4)	7.6 (2.6)
TNNSS, mean (SD)^a^	6.7 (2.6)	6.7 (2.9)	6.4 (2.4)	7.0 (2.7)
SF-12 score, mean (SD)^b^				
Physical health	46.8 (8.2)	48.5 (8.4)	46.1 (8.8)	45.6 (7.6)
Mental health	46.0 (9.9)	43.9 (11.3)	46.5 (9.6)	48.0 (8.3)
High expectations (“very effective”/“effective”) for homeopathy efficacy in SAR, *n* (%)	46 (75.4%)	19 (82.6%)	14 (77.8%)	13 (65.0%)
Expectations for Galphimia glauca in SAR, *n* (%)				
Moderate or high symptom improvement	24 (38.7%)	10 (43.5%)	6 (31.6%)	8 (40.0%)
Small symptom improvement	32 (51.6%)	11 (47.8%)	10 (52.6%)	11 (55.0%)
No improvement	6 (9.7%)	2 (8.7%)	3 (15.8%)	1 (5.0%)
Expectations for individualized homeopathy in SAR, *n* (%)				
Moderate or high symptom improvement	35 (56.5%)	13 (56.5%)	12 (63.2%)	10 (50.0%)
Small symptom improvement	26 (41.9%)	10 (43.5%)	6 (31.6%)	10 (50.0%)
No improvement	1 (1.6%)	0 (0.0%)	1 (5.3%)	0 (0.0%)
Year of recruitment, *n* (%)				
2021	4 (6.5%)	2 (8.7%)	2 (10.5%)	0 (0.0%)
2022	30 (48.4%)	10 (43.5%)	10 (52.6%)	10 (50.0%)
2023	28 (45.2%)	11 (47.8%)	7 (36.8%)	10 (50.0%)
Study centre region, *n* (%)				
Munich	29 (46.8%)	10 (43.5%)	9 (47.4%)	10 (50.0%)
Berlin	33 (53.2%)	13 (56.5%)	10 (52.6%)	10 (50.0%)

a Lower values indicate better status.

b Higher value indicates better status.

SD, standard deviation; SAR, seasonal allergic rhinitis; VAS, visual analogue scale; SF-12, short form quality of live questionnaire; RQLQ, rhinitis quality of live questionnaire; CAM, complementary and alternative medicine; TNSS, total nasal symptom score; TNNSS, total non-nasal symptom score.

A total of 21 study physicians in two hospitals and 11 private outpatient clinics in Berlin (*n* = 33) and Munich (*n* = 29) participated in this trial and treated on average 4.8 patients (median: 4, range 1–13). Change in treatment dose was prescribed in the second consultation in 13 patients (3 in PG, 5 in GG, and 5 in IHG), change in treatment medication in 7 (5 in PG, 1 in GG, and 1 in IHG) patients. High expectations at baseline regarding the effectiveness of homeopathy (effective/very effective) were 75% in the total study population, with higher numbers for expected effectiveness for GG (83%) than for individual homeopathy (78%) and placebo (65%). After 8 weeks a minority of patients in all three groups responded correctly when asked which group they thought they were randomised to (GG: 18%, IHG: 11%, and PG: 19%; Supplementary Table S2).

**Figure 3:**
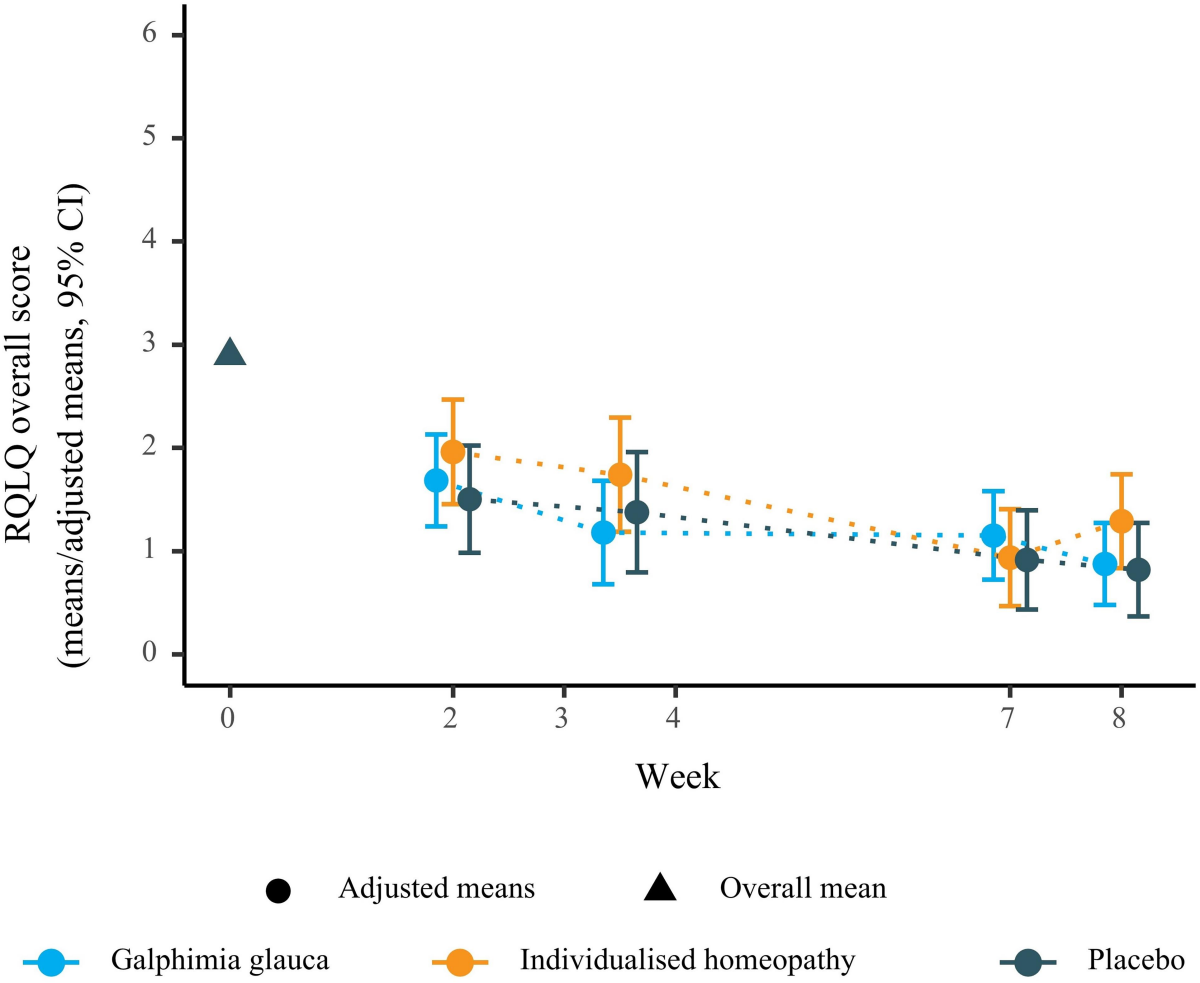
Rhinitis Quality of Life Questionnaire (RQLQ) overall score over time by study group. Adjusted mean RQLQ scores with 95% confidence intervals (CIs) are shown for week 2, the average of weeks 3 and 4, week 7, and week 8. The triangle indicates the overall baseline mean (week 0) across all study participants.

In the analysis of the primary endpoint (mean of RQLQ after weeks 3 and 4), there were no significant group differences (*p* = 0.244) between GG (mean 1.2, 95% CI 0.7-1.7), IHG (1.7, 95% CI 1.2–2.3), and PG (1.4, 95% CI 0.8–2.0) (Figure 3, Table 2). The PP-protocol analysis showed similar results (Supplementary Table S3). The proportion of responders were high in all three groups with 86.4% (GG), 66.7% (IHG), and 81.3% (PG) after 4 weeks [Odds Ratios, 95 CI%: GG vs. PG: 1.46 (0.24, 9.02), *p* = 0.671; IHG vs. PG: 0.46 (0.08, 2.17), *p* = 0.341] (Table 3).

**Table 2:** Primary and secondary outcomes analysed with the FAS. Estimated means and group differences from linear-mixed models, adjusted for respective baseline value (fixed factor) and centre (random factor).

Outcome	Galphimia glauca	Individualised homeopathy	Placebo		Galphimia vs. Placebo		Individualised homeopathy vs. Placebo		Galphimia vs. Individualised homeopathy	
	(*n* = 22)	(*n* = 18)	(*n* = 16)							
	Adjusted Means (95% CI)			*P* Value	Adjusted Differences (95% CI)	*P* Value	Adjusted Differences (95% CI)	*P* Value	Adjusted Differences (95% CI)	*P* Value
RQLQ overall score^a^										
**Mean of weeks 3 and 4** ^b^	**1.2** (**0.7–1.7)**	**1.7** (**1.2–2.3)**	**1.4** (**0.8–2.0)**	**0**.**244**	**−0.2** (**−1.0–0.7)**	**0**.**844**	**0.4** (**−0.5–1.2)**	**0**.**581**	**−0.6** (**−1.4–0.3)**	**0**.**232**
week 2	1.7 (1.2–2.1)	2.0 (1.5–2.5)	1.5 (1.0–2.0)	0.408	0.2 (−0.6–1.0)	0.845	0.5 (−0.4–1.3)	0.392	−0.3 (−1.1–0.5)	0.675
week 3	1.2 (0.8–1.7)	1.8 (1.3–2.3)	1.1 (0.6–1.6)	0.072	0.1 (−0.7–0.9)	0.956	0.7 (−0.1–1.5)	0.119	−0.6 (−1.3–0.2)	0.145
week 4	1.2 (0.6–1.7)	1.7 (1.1–2.3)	1.4 (0.8–2.0)	0.301	−0.2 (−1.1–0.6)	0.776	0.3 (−0.6–1.2)	0.723	−0.5 (−1.4–0.3)	0.282
week 7	1.2 (0.7–1.6)	0.9 (0.5–1.4)	0.9 (0.4–1.4)	0.673	0.2 (−0.5–1.0)	0.719	0.0 (−0.7–0.8)	0.997	0.2 (−0.5–0.9)	0.752
week 8	0.9 (0.5–1.3)	1.3 (0.8–1.7)	0.8 (0.4–1.3)	0.228	0.1 (−0.7–0.8)	0.980	0.5 (−0.3–1.2)	0.282	−0.4 (−1.1–0.3)	0.343
VAS total SAR symptoms^a^										
week 2	42.8 (32.6–53.0)	44.7 (33.0–56.4)	41.8 (29.8–53.8)	0.936	1.0 (−17.5–19.4)	0.991	2.9 (−16.7–22.4)	0.933	−1.9 (−20.0–16.3	0.966
week 4	34.8 (23.0–46.7)	36.2 (22.9–49.4)	37.4 (23.4–51.5)	0.957	−2.6 (−24.0–18.9)	0.955	−1.3 (−23.8–21.3)	0.990	−1.3 (−22.1–19.5)	0.987
week 8	22.5 (12.5–32.5)	23.5 (11.9–35.1)	23.5 (12.0–34.9)	0.987	−1.0 (−18.9–16.9)	0.990	0.1 (−18.8–18.9)	1.000	−1.0 (−19.1–17.1)	0.990
VAS nasal SAR symptoms^a^										
week 2	40.3 (29.1–51.6)	48.8 (35.9–61.6)	45.0 (31.8–58.2)	0.584	−4.6 (−24.9–15.6)	0.845	3.8 (−17.7–25.3)	0.905	−8.4 (−28.4–11.5)	0.567
4	29.0 (16.8–41.2)	35.8 (22.2–49.4)	41.3 (26.8–55.8)	0.387	−12.3 (−34.4–9.8)	0.377	−5.5 (−28.7–17.6)	0.832	−6.8 (−28.2–14.7)	0.726
8	21.5 (10.1–32.9)	20.9 (7.9–34.0)	24.4 (11.6–37.3)	0.907	−2.9 (−23.1–17.2)	0.934	−3.5 (−24.7–17.7)	0.915	0.6 (−20.1–21.2)	0.998
VAS eye SAR symptoms^a^										
week 2	36.7 (25.0–48.4)	37.8 (24.6–51.0)	33.6 (20.1–47.1)	0.876	3.2 (−16.6–22.9)	0.922	4.2 (−16.6–25.0)	0.876	−1.1 (−20.5–18.3)	0.990
week 4	30.3 (18.5–42.1)	29.1 (16.0–42.3)	36.4 (22.5–50.3)	0.683	−6.1 (−27.1–14.8)	0.762	−7.3 (−29.1–14.5)	0.700	1.2 (−19.0–21.3)	0.989
week 8	22.4 (12.1–32.6)	20.3 (8.5–32.1)	19.1 (7.3–30.8)	0.903	3.3 (−15.1–21.7)	0.902	1.2 (−17.9–20.4)	0.986	2.0 (−16.5–20.5)	0.961
VAS respiratory SAR symptoms^a^										
week 2	20.5 (12.0–29.1)	25.1 (15.3–34.9)	10.7 (0.7–20.8)	0.091	9.8 (−5.6–25.2)	0.285	14.3 (−1.9–30.6)	0.094	−4.5 (−19.8–10.7)	0.752
week 4	13.3 (5.1–21.6)	28.2 (19.0–37.4)	7.5 (−2.3–17.3)	0.003	5.8 (−9.1–20.8)	0.614	20.7 (5.1–36.3)	0.007	−14.9 (−29.3 to −0.4)	0.043
week 8	11.0 (2.6–19.3)	17.8 (8.1–27.5)	8.8 (−0.8–18.3)	0.334	2.2 (−12.7–17.1)	0.931	9.0 (−6.6–24.7)	0.349	−6.8 (−21.9–8.2)	0.522
VAS oral SAR symptoms^a^										
week 2	15.9 (5.8–26.0)	26.0 (14.5–37.6)	22.2 (10.4–34.1)	0.372	−6.3 (−24.5–11.9)	0.681	3.8 (−15.4–23.0)	0.882	−10.1 (−28.0–7.8)	0.365
week 4	13.8 (3.7–24.0)	23.6 (12.3–34.9)	17.4 (5.4–29.5)	0.404	−3.6 (−22.0–14.8)	0.886	6.2 (−13.0–25.3)	0.716	−9.8 (−27.5–8.0)	0.385
week 8	8.6 (0.7–16.4)	16.3 (7.3–25.3)	10.1 (1.1–19.1)	0.374	−1.5 (−15.5–12.5)	0.963	6.2 (−8.5–20.8)	0.564	−7.7 (−21.8–6.4)	0.390
TNSS^a^										
week 2	5.3 (4.1–6.5)	5.4 (4.1–6.8)	4.6 (3.2–6.0)	0.572	0.7 (−1.3–2.6)	0.677	0.8 (−1.2–2.8)	0.591	−0.1 (−2.0–1.7)	0.982
week 3	3.8 (2.6–5.0)	5.1 (3.8–6.4)	3.9 (2.4–5.3)	0.223	−0.0 (−2.2–2.1)	0.999	1.2 (−0.9–3.4)	0.359	−1.3 (−3.3–0.7)	0.268
week 4	3.4 (2.1–4.7)	4.6 (3.2–6.0)	4.6 (3.1–6.1)	0.321	−1.2 (−3.6–1.2)	0.442	0.0 (−2.4–2.5)	>0.999	−1.2 (−3.5–1.0)	0.397
week 7	3.2 (2.1–4.3)	3.3 (2.1–4.5)	4.0 (2.8–5.3)	0.477	−0.9 (−2.7–1.0)	0.487	−0.7 (−2.6–1.2)	0.639	−0.2 (−2.0–1.6)	0.968
week 8	2.7 (1.6–3.8)	4.2 (3.0–5.4)	2.9 (1.7–4.2)	0.132	−0.2 (−2.1–1.7)	0.964	1.3 (−0.7–3.2)	0.279	−1.5 (−3.4–0.4)	0.160
TNNSS^a^										
week 2	3.8 (2.5–5.1)	4.3 (2.9–5.8)	4.0 (2.6–5.5)	0.843	−0.2 (−2.5–2.1)	0.968	0.3 (−2.1–2.8)	0.947	−0.5 (−2.8–1.7)	0.831
week 3	3.3 (2.1–4.5)	4.3 (3.0–5.6)	3.6 (2.2–5.0)	0.422	−0.3 (−2.2–1.7)	0.945	0.7 (−1.3–2.7)	0.673	−1.0 (−2.8–0.9)	0.411
week 4	3.3 (2.0–4.5)	3.9 (2.5–5.3)	3.9 (2.5–5.4)	0.682	−0.6 (−2.7–1.5)	0.743	−0.0 (−2.2–2.2)	>0.999	−0.6 (−2.6––1.4)	0.747
week 7	2.8 (1.8–3.9)	2.5 (1.3–3.7)	2.4 (1.2–3.6)	0.813	0.4 (−1.4–2.3)	0.823	0.1 (−1.8–2.0)	0.991	0.3 (−1.5–2.2)	0.887
week 8	2.1 (1.0–3.1)	3.4 (2.2–4.6)	2.1 (1.0–3.3)	0.159	−0.1 (−1.9–1.7)	0.993	1.2 (−0.7–3.2)	0.276	−1.3 (−3.2–0.5)	0.205
SF−12 score, physical component ^c^										
week 2	50.7 (47.4–53.9)	50.0 (46.5–53.6)	51.5 (47.9–55.1)	0.819	−0.9 (−6.5–4.8)	0.928	−1.5 (−7.4–4.3)	0.806	0.7 (−5.0–6.3)	0.957
Week 4	51.5 (48.0–55.0)	49.0 (45.1–52.8)	52.3 (48.2–56.4)	0.322	−0.8 (−6.3–4.7)	0.935	−3.3 (−9.1–2.4)	0.351	2.5 (−2.7–7.8)	0.484
Week 8	54.2 (51.9–56.4)	49.6 (47.0–52.1)	54.0 (51.5–56.6)	0.005	0.1 (−3.7–3.9)	0.996	−4.5 (−8.4 – −0.5)	0.023	4.6 (0.8–8.5)	0.015
SF−12 score, mental component^c^										
week 2	49.1 (46.1–52.2)	46.2 (42.8–49.6)	48.3 (44.7–51.8)	0.397	0.9 (−4.6–6.3)	0.921	−2.1 (−7.47–3.6)	0.655	2.9 (−2.4–8.3)	0.386
Week 4	49.2 (45.7–52.7)	48.4 (44.5–52.3)	49.4 (45.1–53.7)	0.925	−0.1 (−6.6–6.3)	0.998	−1.0 (−7.7–5.7)	0.935	0.8 (−5.3–6.9)	0.943
Week 8	49.9 (46.6–53.1)	50.9 (47.1–54.6)	48.8 (45.0–52.5)	0.692	1.1 (−4.8–7.0)	0.892	2.1 (−4.0–8.2)	0.674	−1.0 (−6.9–4.8)	0.905

a Lower values indicate better status.

b Primary endpoint.

c Higher value indicates better status.

Bold values indicate the primary endpoint.

CI, confidence interval; FAS, Full data set population; RQLQ, rhinitis quality of life questionnaire; SAR, seasonal allergic rhinitis; VAS, visual analogue scale; TNSS, total nasal symptom score; TNNSS, total non-nasal symptom score; SF-12 Short Form—12 Health Survey.

**Table 3:** Responder analysis for RQLQ overall score (mean of weeks 3 and 4). Responder is defined as an improvement from baseline by at least 0.5 points. Results from logistic regression models are presented, both unadjusted and adjusted for RQLQ baseline value and study centre.

	**Unadjusted**		**Adjusted**	
	**Responder, n/N (%)**	**Odds Ratio (95% CI)**	**Odds Ratio (95% CI)**	***p*-value**
Galphimia glauca	19/22 (86.4%)	1.46 (0.24–9.02)	1.63 (0.18–15.98)	0.653
Individualized homeopathic treatment	12/18 (66.7%)	0.46 (0.08–2.17)	0.24 (0.02–1.67)	0.181
Placebo	13/16 (81.3%)	Ref.	Ref.	–

There were no relevant differences between the secondary outcome parameters TNSS, TNNSS, and VAS total symptoms between the three groups after 4 weeks (Table 2). Subgroup analyses showed differences in the RQLQ overall score (mean of weeks 3 and 4) by baseline SAR severity, sex, and treatment expectation, but these may be due to small sample sizes and should be interpreted with caution (Supplementary eTable S3).

Until week four, cetirizine 10 mg was used as rescue medication by 22.7% of patients in the GG, 50.0% in the IHG, and 33.3% in the PG, the corresponding results for cetirizine 20 mg were 0.0%, 5.6%, and 6.7%, and for prednisolone was 4.5%, 16.7%, and 6.7% respectively. Overall, rescue medication was used by 21.7% of patients in the GG, 55.6% in the IHG, and 29.4% in the PG with the largest difference observed between IHG and PG (OR = 3.0, 95% CI 0.8–13.0, *p* = 0.123) (Table 4). In addition, we found no major differences after 8 weeks in most additional secondary outcome parameters.

**Table 4:** *Post-hoc* analysis of rescue medication (RM) use in weeks 1 to 4. RM use is defined as any cetirizine or prednisolone intake during the 4 weeks. Results from logistic regression models are presented, both unadjusted and adjusted for study centre.

	**Unadjusted**	**Adjusted**		
	**RM uses in weeks 1–4, n/N (%)**	**Odds Ratio (95% CI)**	**Adjusted Odds Ratio (95% CI)**	***P* value**
Galphimia glauca	5/23 (21.7%)	0.67 (0.15–2.88)	0.65 (0.15–2.91)	0.577
Individualised homeopathic treatment	10/18 (55.6%)	3.00 (0.77–13.00)	3.36 (0.74–15.16)	0.116
Placebo	5/17 (29.4%)	Ref.	Ref.	–

During the study period, no serious AE occurred. Eight patients (four in the GG, two in the IHG and two in the PG) reported a total of eight mild AE. Most of the AE were eye symptoms such as eye itching and tearing (one in the GG and one in the IHG), the other AE (dry mucous membranes, aggravation in neurodermatitis in PG; severe fatigue, mild gastrointestinal problems, and dry mouth in the GG group) were mentioned once. None of the AE led to clinically relevant disease or was treated in hospital.

## Discussion

### Summary

In this randomised trial, standardised homeopathy with Galphimia glauca and individualised homeopathy were not superior compared to placebo regarding disease-specific quality of life and AR symptoms after 3 and 4 weeks in SAR patients receiving individualised homeopathic case taking. The PP-protocol analysis showed similar results. Results of the secondary parameters confirmed these results. In all three groups we observed high proportions of responders. The validity of the study is limited because the target sample size was not reached.

### Strength and limitations

To our best knowledge this is the first three-armed trial including a standardised and an individualised homeopathic treatment arm compared to a placebo group in SAR. The most relevant strength of this study design was that all three groups received individualised homeopathic case taking so that the effects of homeopathic medication vs. placebo could be investigated. As all groups received IHCT, non-specific effects may have contributed to the observed outcomes and reduced the ability to detect medication-specific effects. Further strengths were the double-blind setting, internationally accepted outcome parameters for SAR, inclusion and exclusion criteria following a high-level published study (20), and well-trained study physicians with many years of homeopathy experience.

The most important limitation of this study was that, despite substantial efforts in recruitment, we had to end the study with only approximately a quarter of the patient numbers within the third recruitment year. The recruitment difficulties are most likely due to the fact that this study took place during the COVID-19 pandemic. In our view, an additional reason was that interested patients were not willing to participate in this study because of the high amount of documentation required, and possibly due to the controversial debate of homeopathy in German media. As a result, we were only able to include approximately 5% of the screened patients in the study, a fact that also limits the external validity of this study. In summary, only 62 of the planned 270 participants could be recruited. Consequently, the study was underpowered, and the observed between-group estimates, together with their wide confidence intervals, should be interpreted with caution. In our previous study on acupuncture in SAR with almost the same exclusion criteria, we recruited close to 20% of the screened patients (20). In addition, due to participants being recruited primarily through media, they may not necessarily be representative of SAR patients in general. This is underlined by the relatively high expectation rate of 76% those patients had towards the effectiveness of homeopathic treatment in this study. Given the small sample size, some baseline imbalances between groups remained after randomisation, including differences in age, sex distribution, prior medication use, and treatment expectations, which may have influenced the results. In addition, SAR patients may have had less allergen contact during the pandemic due to the use of masks inside and outside of closed rooms because of lock-down periods and increased opportunities for home office. This may also have contributed to the high response rate in all three groups as well as the regression to the mean by the natural course of the disease (about half of the patients were included in June and July in the SAR year). Seasonal variation in pollen exposure may also have influenced symptom severity and treatment responses. However, recruitment months were broadly balanced across the three groups, making major differences in pollen exposure between groups unlikely. Nevertheless, residual confounding due to variations in environmental allergen exposure cannot be excluded.

In addition, the high baseline expectations regarding a potential success of homeopathic treatment may have contributed to placebo responses and treatment expectation effects. Although rescue medication use was recorded and permitted equally across groups, its use was numerically higher in the individualised homeopathy group. As no statistical adjustment for rescue medication use was performed, a possible influence on symptom-related outcomes cannot be excluded. The higher use of rescue medication may also indicate a greater symptom burden in the individualised homeopathy group.

### Comparison with other studies/evidence

One important aim of this study was to test the efficacy of GG because there was positive evidence from a meta-analysis by Lüdtke and Wiesenauer including eleven trials (seven randomised, double-blind, placebo-controlled trials and four non-placebo-controlled trials) that showed GG to be superior to placebo (12). However, the authors of this former meta-analysis concede that the analyses of the studies were not carried out according to the intention-to-treat approach, but according to the per-protocol approach. In addition, a more precise analysis of the study quality was not carried out. In contrast to all other studies performed before (four by the same first author), the overall quality of our trial methods is higher including a pre-published protocol, a predefined statistical analysis plan, triple blinding of patients, study physicians and study team. Interestingly, the cited meta-analysis already showed high placebo success rates of between 50% and 79%, the corresponding success rates for Galphimia were max. 88%. However, only two studies showed a statistically significant difference between verum and placebo (12). These responder results again match the results of our study with overall high but comparable response rates of Galphimia glauca and placebo.

The second aim of this study was to investigate the efficacy of individualised homeopathic medication in SAR. This is the classic homeopathic treatment strategy, in which the substance to be prescribed is selected and administered individually. Although this approach is the most common homeopathic treatment strategy used by study physicians, results of a meta-analysis by Banerjee (13) show, that it has not been tested for AR yet in a high-quality randomised controlled clinical trial. Although two relatively small prospective observational studies showed clinical relevant effects of individualised homeopathy in allergic diseases (23, 24), the interpretation of the success rates of the prescribed homeopathic drugs is severely limited due to the fact that these studies have no control groups and a high risk of bias. In our study the proportion of responders in the individualised homeopathy group was slightly lower compared to the placebo and the GG group, but the difference was not statistically significant. Given the small sample size and remaining baseline imbalances between groups, these differences should be interpreted with caution and may partly reflect random variation. The high response rates of placebo and both homeopathic treatments, which are even higher than in the acupuncture trial before (20), suggest that factors other than the study medication itself may have contributed to the observed results. It is possible that the high response rates were influenced by several factors such as whole treatment setting, comprised of an individualised and lengthy homeopathic consultation process, the physician-patient interaction, the outcome expectation, and by the regression to the mean by the natural course.

One theory would be that homeopathy does not work through the medication administered, rather through the homeopathic consultation process, and this has been previously expressed (5). This hypothesis had already been formulated after an earlier randomised trial in rheumatoid arthritis, in which the effect of homeopathy was located in the consultation process, rather than the homeopathic drug 25). In our study, all three treatment groups received a detailed homeopathic interview, an individualised case analysis, and repertorisation. However, the homeopathic interview may not only be significant due to its long duration, but also due to its special characteristics: it is usually conducted in an empathic non-judgemental manner, the therapist listens carefully to the patient, if possible without interrupting them, is interested in the exact details of the presenting complaints, and only then concretises and supplements the information with guided interview questions. Mental and emotional symptoms are also enquired about, so that the entire consultation takes place in a bio-psycho-social setting and can last up to 120 min. In the qualitative study of the study on rheumatoid arthritis it was found that the homeopathic consultation helped patients to better cope by facilitating either improved physical health, well-being, and/or coping with illness (26). However, we did not include a “no-treatment” or “conventional medicine only” control group which could have helped to assess the effect of the specific homeopathic consultation process vs. the conventional approach. Our hypothesis is that the individualised homeopathic case taking process has effects which may be comparable to standard anti-allergic medication due to the unspecific mechanisms which were described above. In a large RCT study in irritable bowel syndrome, it was demonstrated that non-specific effects can produce statistically and clinically significant outcomes and the patient-practitioner relationship was the most robust component (27).

Over the last two decades, several meta-analyses of randomised controlled trials were published with contradictory results for the efficacy of homeopathy depending on the study methodology used [e.g., Linde et al. (8), Shang et al. (9), Mathie et al. (10) and Hamre et al. (11)]. Considering that our three-arm trial included an individualised homeopathic case taking in all groups; to investigate the specific effect of homeopathic medication, our study is not comparable to other studies which were included in these meta-analyses.

### Further research

We are of the opinion that our three-armed approach, in which both individualised and standardised homeopathy were tested against placebo in an otherwise equal setting, adds markedly to the discussion concerning the most appropriate method of research of homeopathy. However, it could have been interesting if, in addition to the three groups, a further group receiving no individualised homeopathy case taking but a conventional treatment with antihistamines of the second or third generation was to be included. Future studies may also investigate other homeopathic medicines commonly used for seasonal allergic rhinitis using a comparable randomised controlled design. In addition, longer intervention and follow-up periods may help to better assess potential long-term effects of homeopathic treatment.

## Conclusion

Standardised and individualised homeopathic drugs were not superior compared to placebo suggesting that treatment response was not based on study medication. The validity of the study and its conclusions are limited by the fact that the recruitment target was not achieved.

## Data Availability

The raw data supporting the conclusions of this article will be made available by the authors, without undue reservation.
