# Supplementary material for: Homeopathic medication for seasonal allergic rhinitis—a randomised placebo-controlled trial
Source: Front Allergy. 2026 Jul 17;7:1815934. doi: 10.3389/falgy.2026.1815934 (PMC13424193; doi:10.3389/falgy.2026.1815934)
Supplement: Supplementary file 1 [file Table1.docx]

**SUPPLEMENT**

**eTable 1**: Blinding procedure - participants answers regarding the questions in which group they may have been randomised after 8 weeks.

**eTable 2**: HOMEOSAR study - Recruitment of patients in the SAR - seasons

**eTable 3:** Sensitivity analyses for primary and secondary outcomes

Supplement

eTable 1: Blinding procedure – participants’ answers to the question in which group they may have been randomized (assessed after 8 weeks).

|  | **Galphimia glauca** | **Individual** | **Placebo** |
| --- | --- | --- | --- |
|  | N = 22 | N = 18 | N = 16 |
| **Blinding** | n (%) | n (%) | n (%) |
| Galphimia glauca | 4 (18.2%) | 4 (22.2%) | 4 (25.0%) |
| Individualized homeopathic treatment | 8 (36.4%) | 2 (11.1%) | 4 (25.0%) |
| Placebo | 2 (9.1%) | 8 (44.4%) | 3 (18.8%) |
| Don’t know | 8 (36.4%) | 4 (22.2%) | 5 (31.3%) |

eTable 2: HOMEOSAR study – patients´ recruitment in the SAR – months 2021-2023

| **Recruitment Month** | **Total (N=62)** | **Galphimia glauca**  **(N=23)** | **Individualized homeopathic treatment**  **(N=19)** | **Placebo**  **(N=20)** |
| --- | --- | --- | --- | --- |
| March | 4 (6.5%) | 1 (4.3%) | 1 (5.3%) | 2 (10.0%) |
| April | 11 (17.7%) | 3 (13.0%) | 4 (21.1%) | 4 (20.0%) |
| May | 19 (30.6%) | 8 (34.8%) | 6 (31.6%) | 5 (25.0%) |
| June | 18 (29.0%) | 8 (34.8%) | 6 (31.6%) | 4 (20.0%) |
| July | 10 (16.1%) | 3 (13.0%) | 2 (10.5%) | 5 (25.0%) |

SAR = Seasonal Allergic Rhinitis

eTable 3: Sensitivity analyses for primary and secondary outcomes and subgroup analyses.

|  |  |  | **Galphimia glauca** | **Individualized homeopathy** | **Placebo** |  | **Galphimia glauca vs. Placebo** | | **Individualized homeopathy vs. Placebo** | | **Galphimia glauca vs. Individualized homeopathy** | |
| --- | --- | --- | --- | --- | --- | --- | --- | --- | --- | --- | --- | --- |
| **Outcome** | **Patient set** | N | Adjusted Means (95% CI) | | | P Value | Adjusted Differences  (95% CI) | P Value | Adjusted Differences  (95% CI) | P Value | Adjusted Differences  (95% CI) | P Value |
| RQLQ overall score*, mean of weeks 3 and 4 | PP | 41 | 1.0 (0.5 - 1.5) | 2.0 (1.4 - 2.5) | 1.1 (0.5 - 1.7) | 0.012 | -0.1 (-1.0 - 0.8) | 0.949 | 0.9 (-0.1 - 1.8) | 0.086 | -1.0  (-1.9 - -0.1) | 0.023 |
| RQLQ overall score*, mean of weeks 3 and 4 | PP, no prednisolone | 38 | 1.1 (0.6 - 1.5) | 1.7 (1.1 - 2.3) | 1.1 (0.5 - 1.6) | 0.150 | -0.0 (-0.9 - 0.8) | 0.996 | 0.6 (-0.3 - 1.5) | 0.278 | -0.6  (-1.5 - 0.2) | 0.198 |
| RQLQ overall score*, mean of weeks 3 and 4 | ITT (missing values imputed) | 62 | 1.2 (0.7 – 1.7) | 1.8 (1.2 – 2.3) | 1.4 (0.8 -2.1) | 0.572 | -0.2 (-0.9 - 0.5) | 0.533 | 0.3 (-0.4 - 0.8) | 0.404 | -0.5  (-1.2 - 0.2) | 0.126 |
|  | | *Model with study region instead of center as random factor:* | | | | | | | | |  |  |
| RQLQ overall score*, mean of weeks 3 and 4 | FAS | 56 | 1.2 (0.4 - 2.0) | 1.8 (1.0 - 2.5) | 1.4 (0.6 - 2.1) | 0.273 | -0.2 (-1.0 - 0.7) | 0.867 | 0.4 (-0.5 - 1.3) | 0.590 | -0.5  (-1.4 - 0.3) | 0.257 |
|  | | *Subgroup analyses for* RQLQ overall score, mean of weeks 3 and 4 | | | | | | | | |  |  |
| By VAS SAR symptom severity* |  | 56 |  |  |  | P value for interaction |  |  |  |  |  |  |
| moderate (n=28) | FAS |  | 1.7 (0.9 - 2.4) | 1.5 (0.8 - 2.2) | 1.6 (0.8 - 2.4) | 0.113 | 0.1 (-1.2 - 1.3) | 0.987 | -0.1 (-1.3 - 1.1) | 0.975 | 0.2 (-1.0 - 1.4) | 0.928 |
| Severe (n=28) | FAS |  | 0.9 (0.2 - 1.5) | 2.1 (1.2 - 2.9) | 1.1 (0.3 - 2.0) |  | -0.3 (-1.5 - 1.0) | 0.861 | 0.9 (-0.4 - 2.3) | 0.232 | -1.2 (-2.4 - -0.0) | 0.048 |
| By Patient’s sex |  | 56 |  |  |  |  |  |  |  |  |  |  |
| Female (n=26) | FAS |  | 1.0 (0.3 - 1.6) | 1.7 (0.9 - 2.5) | 2.2 (1.5 - 3.0) | 0.003 | -1.3 (-2.4 - -0.1) | 0.023 | -0.5 (-1.8 - 0.7) | 0.554 | -0.7 (-1.9 - 0.4) | 0.290 |
| Male (n=30) | FAS |  | 1.4 (0.8 - 2.0) | 1.8 (1.2 - 2.4) | 0.5 (-0.2 - 1.3) |  | 0.9 (-0.2 - 2.0) | 0.151 | 1.3 (0.1 - 2.4) | 0.025 | -0.4 (-1.4 - 0.6) | 0.627 |
| Patient’s expectation for symptom improvement if treatment = Galphimia glauca |  | 56 |  |  |  |  |  |  |  |  |  |  |
| Low (n=34) | FAS |  | 0.8 (0.2 - 1.5) | 2.1 (1.5 - 2.8) | 1.4 (0.7 - 2.1) | 0.018 | -0.5 (-1.6 - 0.6) | 0.488 | 0.8 (-0.3 - 1.9) | 0.226 | -1.3 (-2.4 - -0.2) | 0.015 |
| High (n=22) | FAS |  | 1.6 (0.9 - 2.3) | 1.0 (0.1 - 1.8) | 1.4 (0.5 - 2.4) |  | 0.2 (-1.2 - 1.5) | 0.945 | -0.5 (-2.0 - 1.1) | 0.751 | 0.6 (-0.7 - 2.0) | 0.492 |

* Lower values indicate better status; † Higher value indicates better status. CI = confidence interval. ITT = Intention to treat population, FAS = Full data set population, PP = per protocol population. RQLQ = Rhinitis Quality of Life Questionnaire, SAR = Seasonal Allergic Rhinitis, VAS = visual analogue scale
